# Retrotransposon targeting to RNA polymerase III-transcribed genes

**DOI:** 10.1186/s13100-018-0119-2

**Published:** 2018-04-23

**Authors:** Stephanie Cheung, Savrina Manhas, Vivien Measday

**Affiliations:** 1 Department of Biochemistry and Molecular Biology, Faculty of Medicine, University of British Columbia, Vancouver, BC V6T 1Z4 Canada; 2 Department of Food Science, Wine Research Centre, Faculty of Land and Food Systems, University of British Columbia, Room 325-2205 East Mall, Vancouver, British Columbia V6T 1Z4 Canada

**Keywords:** Retrotransposon, *S. cerevisiae*, Ty element, RNA polymerase III, tRNA, Integrase

## Abstract

Retrotransposons are genetic elements that are similar in structure and life cycle to retroviruses by replicating via an RNA intermediate and inserting into a host genome. The *Saccharomyces cerevisiae* (*S. cerevisiae*) Ty1–5 elements are long terminal repeat (LTR) retrotransposons that are members of the Ty1-*copia* (*Pseudoviridae*) or Ty3-*gypsy* (*Metaviridae*) families. Four of the five *S. cerevisiae* Ty elements are inserted into the genome upstream of RNA Polymerase (Pol) III-transcribed genes such as transfer RNA (tRNA) genes. This particular genomic locus provides a safe environment for Ty element insertion without disruption of the host genome and is a targeting strategy used by retrotransposons that insert into compact genomes of hosts such as *S. cerevisiae* and the social amoeba *Dictyostelium*. The mechanism by which Ty1 targeting is achieved has been recently solved due to the discovery of an interaction between Ty1 Integrase (IN) and RNA Pol III subunits. We describe the methods used to identify the Ty1-IN interaction with Pol III and the Ty1 targeting consequences if the interaction is perturbed. The details of Ty1 targeting are just beginning to emerge and many unexplored areas remain including consideration of the 3-dimensional shape of genome. We present a variety of other retrotransposon families that insert adjacent to Pol III-transcribed genes and the mechanism by which the host machinery has been hijacked to accomplish this targeting strategy. Finally, we discuss why retrotransposons selected Pol III-transcribed genes as a target during evolution and how retrotransposons have shaped genome architecture.

## Background

Genome evolution and plasticity are impacted by endogenous DNA sequences called transposable elements (TEs), that can mobilize within a genome [1]. TEs, which make up a significant portion of eukaryotic genomes, are divided into two classes: class I retrotransposons that mobilize via an RNA intermediate using a “copy and paste” mechanism and class II DNA transposons that use a “cut and paste” mechanism [2, 3]. Class I retrotransposons can be further divided into five orders: LTR-retrotransposons, *DIRS*-like elements, *Penelope*-like elements, long interspersed elements (LINEs) and short interspersed elements (SINEs) [3]. LTR-retrotransposons carry characteristic flanking repetitive sequences and are similar to retroviruses in structure and replication but do not exit the cell. The *S. cerevisiae* genome contains five types of LTR-retrotransposon elements, known as Ty1–5, that transpose through an RNA intermediate and produce intracellular virus-like particles (VLPs) [4, 5]. The majority of the *S. cerevisiae* LTR retrotransposons belong to the *copia* (*Pseudoviridiae*) family (Ty1, 2, 4, 5) whereas Ty3 belongs to the *gypsy* (*Metaviridae*) family [5]. Ty1–4 elements enter the genome in the vicinity of Pol III-transcribed genes, whereas Ty5 elements insert into silent chromatin [5, 6] .

**Fig. 1:**
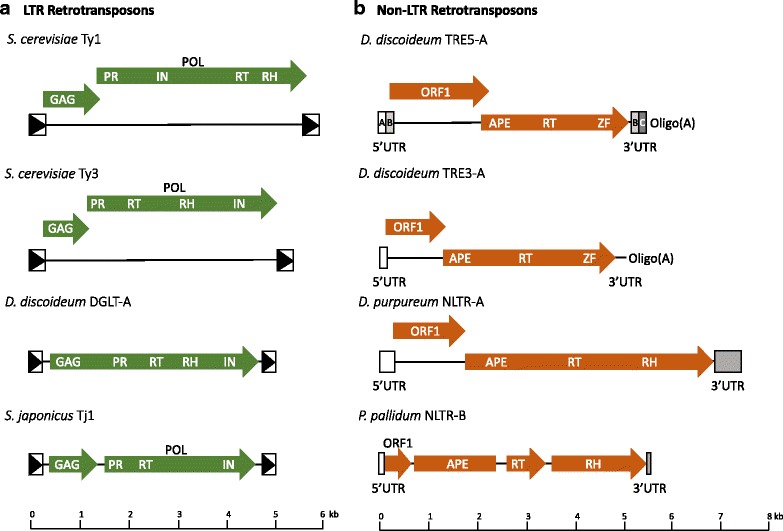
LTR and non-LTR retrotransposons that target to tRNA genes. **a.** LTR retrotransposons. Ty1, Ty3, DGLT-A and Tj1 elements are depicted in dark green. The boxed black arrows represent the LTRs flanking the two ends of the elements. The first ORF of the Ty1 element encodes Gag and the second ORF encodes a polypeptide (Pol) which is further processed into protease (PR), integrase (IN), and reverse transcriptase (RT)/ ribonuclease H (RH). Ty3 differs in structure from Ty1 by swapping positions of IN and RT/RH. For both Ty1 and Ty3, the Pol polypeptide is generated by a + 1 translational frameshift 38 bp upstream of the 3’end of Gag [169–171]. The *D. discoideum* DGLT-A element contains one ORF that encodes for both Gag and Pol proteins. DGLT-A belongs to the Ty3-*gypsy* clade, signified by the arrangement of *pol* with IN after RT/RH [172]. *S. japonicas* Tj1 has a similar structural arrangement as Ty3 with *GAG * and *POL* as two separate ORFs. The *GAG* ORF has a stop codon that is thought to be translationally suppressed to allow for translation of the *POL* ORF which lacks a start codon [121]. The length of each element is depicted by the scale at the bottom in kb. **b.** non-LTR retrotransposons. *D. discoideum* TRE5-A and TRE3-A, *D. purpureum* NLTR-A and *P. pallidum* NLTR-B are depicted in dark orange and all share a similar structural arrangement. All elements except NLTR-B have two ORFs flanked by untranslated regions (UTR), with TRE5-A and TRE3-A ending with an oligo(A) tail. The 5′ and 3’UTR of TRE5-A are arranged into A- and B-modules, and B- and C-modules respectively. The protein domain arrangement of TRE5-A and TRE3-A ORF2 is the same and encodes a protein containing an apurinic/apyrimidinic endonuclease (APE), RT, and zinc-finger (ZF) domain. Both TRE5-A and TRE3-A require a − 1 frameshift for translation of ORF2 [137, 173]. NLTR-A and NLTR-B have a similar arrangement to the TRE5-A and TRE3-A elements except that an RH domain substitutes for the ZF domain. In addition, NLTR-B has three separate ORFs for APE, RT and RH. It is not yet known if the 5′ and 3’ UTRs of NLTR-A and NLTR-B are arranged into modules. NLTR-A ORF1 overlaps with ORF2 by 13 bp but whether a frameshift occurs or not for translation of ORF2 is not yet known [124]. NLTR-B does not contain overlapping ORFs, however RT does not contain a start codon [124]. The length of each element is depicted by the scale at the bottom in kb

Thirty-two copies of the Ty1 element, which is the most abundant *S. cerevisiae* TE, as well as 279 solo LTRs, are present in the genome of the commonly used laboratory strain S288C. Ty1 elements are 5.9 kb in length and composed of *GAG* and *POL* open reading frames (ORFs) sandwiched in-between 334 bp LTR sequences [7, 8]. *GAG* encodes the structural protein of the VLP, while *POL* produces a polyprotein of protease (PR), IN, reverse transcriptase (RT) with ribonuclease H activity (RH) (Fig. 1) [7]. The *copia* and *gypsy* families differ in the order of RT/RH and IN such that the Ty3-*gypsy* element has RT/RH followed by IN (Fig. 1) [9]. Ty1 replication begins with transcription of a genomic Ty1 element using the host RNA Pol II machinery, translation of the Ty1 messenger RNA (mRNA) into the Gag protein or the Gag-Pol fusion protein when a + 1 ribosomal frameshift event places Gag and Pol in frame [7]. The Gag and Pol polypeptide, an initiator methionine tRNA (tRNAi^met^) and two Ty1 mRNA transcripts, are assembled into VLPs where Gag and Pol undergo processing and maturation by PR [10–12]. Following RT-mediated reverse transcription of the Ty1 mRNA in the VLPs, a pre-integration complex composed minimally of newly synthesized Ty1 cDNA and IN, called the intasome, is generated. The intasome localizes to the nucleus where IN-mediated insertion of the Ty1 cDNA preferentially occurs in a ~ 1 kb window upstream of genes actively transcribed by RNA Pol III including all 275 nuclear tRNA genes and the 5S ribosomal RNA (rRNA) gene [13, 14]. Ty1 cDNA can also enter the genome via homologous recombination with a pre-existing Ty1 element [15, 16]. When Ty1 insertion assays are performed in vitro using purified VLPs and target DNA, targeting is random suggesting that *S. cerevisiae* host factors are required to target Ty1 elements to Pol III genes [17, 18]. As early as 1979, it was observed that genomic copies of Ty1 are associated with tRNA genes [19]. By 1993, the 5′ region upstream of tRNA genes was defined as the preferred Ty1 element insertion site and the glycine tRNA gene *SUF16* was identified as a Ty1 insertion hotspot [20]. Upon completion of the *S. cerevisiae* genome sequence it was clear that the majority of Ty1–4 elements were located adjacent to tRNA genes or other Pol III-transcribed genes [8, 21]. The Ty2 and Ty4 elements share the same insertion preference as Ty1 elements, whereas the Ty3 element integrates specifically at the RNA Pol III transcription start site (TSS) [5]. To understand the mechanism of Ty insertion at tRNA genes, it is important to briefly describe the RNA Pol III transcription machinery.

## RNA Pol III transcription machinery

RNA Pol III is a 17-subunit complex that, along with TFIIIB and TFIIIC transcription complexes, transcribes all tRNAs, and other essential RNAs including the U6 small nuclear RNA [22, 23]. The 5S rRNA gene, which is also transcribed by RNA Pol III requires the additional TFIIIA transcription factor. For the purposes of this review, we briefly describe tRNA gene promoters because of the frequent use of tRNA genes in Ty1 studies. tRNA genes contain an internal promoter with two highly conserved sequence elements, a proximal box A and a more distal box B, within the transcribed region. tRNA gene activation first requires association of TFIIIC with DNA, followed by TFIIIB, which then recruits RNA Pol III [22, 23]. TFIIIC is a 6-subunit complex with a τA subcomplex that recognizes box A and a τB subcomplex that recognizes box B [24, 25]. TFIIIB is assembled from three proteins in yeast – Brf1, TATA binding protein (TBP)/Spt15 and Bdp1 [26]. Brf1 and TBP assemble first into the transcription complex followed by interaction with Bdp1 [27]. Once TFIIIB is bound, the RNA Pol III transcription complex can assemble onto the promoter [28]. The common features of all types of RNA Pol III promoters is that TFIIIC, TFIIIB and RNA Pol III are recruited to activate transcription. Mutation of the *SUF16* tRNA promoter, such as a point mutation in box B, that severely reduces transcription, also dramatically reduces Ty1 element insertion suggesting that active Pol III transcription is required for Ty1 transposition [17].

## Mechanism for Ty1 insertion upstream of Pol III-transcribed genes

Two reports have demonstrated that Pol III subunits are essential host factors required for Ty1 intasome targeting upstream of Pol III-transcribed genes [29, 30]. Below we outline the data presented in each study that supports a role for Pol III as the Ty1-IN host factor.

Cheung et al. overexpressed the Ty1 element from an inducible plasmid in yeast cells, purified Ty1-IN using the 8b11 monoclonal anti-IN antibody, then performed mass spectrometry (MS) to identify Ty1-IN co-purifying proteins [18, 30]. Five RNA Pol III subunits were identified by MS (Rpc25, 34, 40, 53, 82) that co-purified with Ty1-IN from two independent purifications [30]. The 17-subunit RNA Pol III complex consists of a ten-subunit core with five subunits shared with all three Pols (Rpb5, Rpb6, Rpb8, Rpb10, Rpb12) and two others shared between Pol I and III (Rpc40 and Rpc19) [31]. The seven remaining subunits are the Rpc53/37 heterodimer, which is the structural counterpart of TFIIF, the Rpc82/34/31 heterotrimer which is related to TFIIE and the Rpc25/17 dimer that is similar to Rpb4/7 [31]. GFP-tagged versions of the two largest subunits of RNA Pol III (Rpc1 and Rpc2) co-purified with Ty1-IN but the homologous Pol II subunits (Rpb1 and Rpb2, respectively) did not, suggesting that Ty1-IN specifically interacts with the Pol III complex [30]. Pol III subunits tagged with either GFP or HA were purified from yeast lysates and Rpc17, 19, 25, 34, 53, and 82 all co-purified with Ty1-IN. However, since the Pol III complex is intact during these pull-downs, it is not possible to pinpoint which Pol III subunit interacts directly with Ty1-IN using this method. Therefore, in vitro binding experiments were also performed and demonstrated that Rpc31, 34 and 53 can interact directly with Ty1-IN using bacterially expressed proteins [30].

There are a few pieces of evidence to support the hypothesis that the Rpc53/37 heterodimer may be directly involved in targeting Ty1-IN. Removal of the N-terminal 280 amino acids from Rpc53 (*rpc53Δ2–280*) significantly reduced Ty1 element targeting upstream of the *SUF16* gene [30]. However, Ty1 mobility in the *rpc53Δ2–280* mutant was not significantly impaired (~ 75% of wild type levels) suggesting that the Ty1 element may be targeted elsewhere in the genome. When GFP pull-down experiments were performed with Rpc37-GFP in the *rpc53Δ2–280* strain background, Ty1-IN no longer co-purified with Rpc37 [30]. As well, a V5-tagged version of rpc53D2-280 does not interact with Ty-IN in yeast lysates (S.C. and V.M. unpublished data). Since Rpc82-GFP, Rpc19-GFP and Rpc17-GFP interact with Ty1-IN in the rpc53D2-280 mutant, the defect in Ty1 targeting may be due to a loss of interaction between Ty1-IN and the Rpc53/37 heterodimer. However, it is not known which other Ty1-IN and Pol III subunit interactions may be compromised in the *rpc53Δ2–280* mutant.

Bridier-Nahmias et al., discovered an interaction between Ty1-IN and the Rpc40 subunit of RNA Pol III using a yeast two-hybrid assay that was confirmed by co-immunoprecipitation (IP) analysis between HA-tagged Rpc40 and Ty1-IN [29]. Using the yeast two-hybrid method, a specific interaction of Rpc40 was detected with only the C-terminal 57 amino acids of Ty1-IN [29]. Cheung et al. found that removal of 75 amino acids from the C-terminus of Ty1-IN abrogated the interaction of Ty1-IN with Rpc82-GFP in pull-down experiments [30]. Therefore, the data from both groups suggests that the C-terminus of Ty1-IN is important for interaction with Pol III. Interestingly, the C-terminus of Ty5-IN interacts with Sir4 to target Ty5 to silent chromatin [32, 33]. To disrupt the interaction of Ty1-IN with RNA Pol III without reducing Pol III transcription, Bridier-Nahmias et al., made clever use of a previous observation that the *Schizosaccharomyces pombe* (*S. pombe*) Rpc40 subunit (Rpc40sp) can functionally replace the *S. cerevisiae* Rpc40 subunit [34]. When Rpc40 was replaced with Rpc40sp, the interaction with Ty1-IN and Ty1 element targeting upstream of Pol III genes was disrupted [29]. Interestingly, overall Ty1 mobility was not impaired in the Rpc40sp strain and genome-wide mapping revealed that Ty1 elements were preferentially targeted to the last 20-30 kb at the ends of each chromosome [29]. This work reveals that Ty1-IN may interact with alternative host factors in the absence of the Rpc40-Ty1-IN interaction. The Ty5 retrotransposon integrates preferentially into heterochromatin at telomeres and silent mating loci [35–37]. It would be interesting to test if Sir4, which targets Ty5-IN to heterochromatin, is able to interact with Ty1-IN in the absence of Rpc40 [32, 33].

**Fig. 2:**
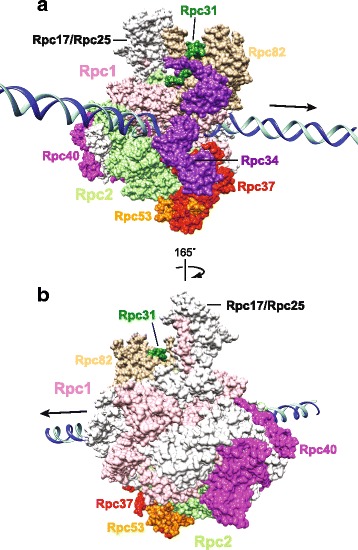
Pol III structure highlighting subunits that may interact with Ty1-IN. The Pol III surface view is based on the cryoelectron microscopy structure of the initially transcribing Pol III complex (Protein Data Bank code 6f41) [44] with TBP, Brf1 and Bdp1 structures excluded. The arrow points to downstream DNA and the DNA template and non-template strands are coloured in light blue and dark blue, respectively. **a** The highlighted Pol III subunits are Rpc31 (dark green), Rpc34 (purple), Rpc82 (beige), Rpc1 (light pink), Rpc2 (light green), Rpc40 (magenta), Rpc53 (orange) and Rpc37 (red). The N-terminus of Rpc53 (amino acids 1–270) is not depicted due to lack of structural data. **b** Same as in (**a**) except rotated 165^o^

Structures of retroviral intasomes, which are INs in complex with their viral cDNA, have revealed that intasomes can be a tetramer, an octamer or even higher order oligomers of IN protomers [38–43]. The structure of Ty1-IN has not been determined yet, nor what type of oligomer structure it may form. Since Ty1-IN is a 636-amino acid protein (predicted molecular weight of 71.5 kDa for a monomer or 286 kDa for a tetramer) it is possible that the Ty1-IN intasome could interact with multiple Pol III subunits as the entire 17-subunit RNA Pol III complex is ~ 690 kDa. In Fig. 2, we provide a structure of RNA Pol III based on recent structural data that highlights the 2 largest Pol III subunits (Rpc1,2) the Pol III specific subunits (Rpc31/34/82 heterotrimer, Rpc53/37 dimer, Rpc17/25 dimer) and Rpc40 [44]. Of the highlighted subunits in Fig. 2, there is evidence that Rpc31, Rpc34, Rpc40 and Rpc53 may interact directly with Ty1-IN [29, 30]. Rpc40 is positioned in the Pol III complex facing the upstream DNA which may be relevant because Ty1 elements are only inserted upstream of Pol III transcribed genes [17, 21]. Future structural studies of Ty1-IN binding to RNA Pol III will help determine precisely how this interaction takes place.

## Ty1 targeting into chromatin

### Chromatin remodeling

Yeast tRNA genes have an open chromatin structure with strongly ordered upstream nucleosomes and a nucleosome-depleted gene body [45–47]. Ty1 element genome-wide mapping studies demonstrated that Ty1 insertions are targeted to two DNA sites on the same surface of the nucleosome at the H2A/H2B interface [13, 14, 48]. Structural studies of the prototype foamy virus (PFV) intasome, a homotetramer of PFV-IN, attached to a nucleosome have revealed striking similarity to the nucleosome data from the Ty1 genome-wide mapping studies [40, 49]. The PFV intasome also interacts with one H2A/H2B heterodimer and two DNA strands on the same surface of the nucleosome [49]. Therefore, the interaction between homotetramer INs and nucleosomes may be conserved.

Chromatin remodeling complexes, which utilize ATP to mobilize nucleosomal DNA, impact Ty1 transcription and Ty1 genome integration. The SWI/SNF and SAGA chromatin-remodeling complexes are required for Ty1 transcription whereas Isw1 and Isw2 (catalytic subunits of three ISW1 chromatin remodeling enzymes) inhibit Ty1 transcription [50–53]. Deletion of Isw2 disrupts the periodic Ty1 integration pattern upstream of tRNA genes likely because Isw2 is needed to maintain the nucleosome array upstream of all tRNA genes [46, 54, 55]. Isw2 may be recruited by Bdp1, a component of TFIIIB, because removal of the Bdp1 N-terminus (*bdp1-Δ240*) also results in altered nucleosome positioning and Ty1 insertion upstream of tRNA genes [54]. However, Ty1 elements are still targeted to tRNA genes in the *bdp1-Δ240* mutant strain and Bdp1 does not interact with Ty1-IN in yeast lysates [30, 54]. This data suggests that the TFIIIB complex is not a Ty1-IN host targeting factor.

Structural maintenance of chromosomes (Smc) complexes that are essential for chromosome condensation and segregation localize to Pol III-transcribed genes. The Smc2/4 condensin complex, which is required for chromosome compaction, binds to tRNA genes and physically interacts with TFIIIB and TFIIIC [56, 57]. A potential role for condensin in Ty1 targeting has not yet been explored. The Smc1/3 cohesin complex, which holds sister-chromatids together, requires the Scc2/4 complex to load onto chromosomes [58, 59]. Notably, Scc2/4 binds to the same chromosomal locations as condensin and may be recruited by TFIIIC to bind box B sites [56]. Once cohesin loads onto chromosomes at Scc2/4 binding sites, it relocalizes to sites of active transcription [60]. The separation of sister chromatids in mitosis requires cleavage of the cohesin ring by a conserved cysteine protease called separase, or Esp1 in yeast [61]. Interestingly, Esp1 was found to physically interact with Ty1-IN and this interaction is enriched in metaphase cells [62]. An *esp1–1* mutant with reduced cleavage activity has reduced Ty1 mobility and Ty1 insertion upstream of the *SUF16* tRNA gene [62]. Consistently, mutations in cohesin proteins (including Scc1 which is cleaved by Esp1) cause enhanced Ty1 mobility and increased Ty1 element insertion upstream of the *SUF16* tRNA gene [62]. The simplest interpretation of why increased Ty1 mobility is observed upon removal of the cohesin complex is that the Ty1 intasome has increased access to nucleosomes. However, the physical interaction between Ty1-IN and Esp1 could be one mechanism by which Ty1-IN is targeted to chromatin [62].

### Histone modification

Chromatin-modifying enzymes, which add or remove post-translational modifications to the core histones, also impact Ty1 targeting. Hos2 and Set3, which are both members of the Set3 histone deacetylase complex, are required for the efficient integration of Ty1 elements upstream of tRNA genes [63]. Although Hos2 is required for Ty1 integration, genome-wide Ty1 mapping studies did not find any difference in the Ty1 insertion pattern of a *hos2Δ* mutant compared to a wild type strain [13]. Deletion of the Rpd3 histone deacetylase caused reduced Ty1 insertion upstream of the *SUF16* tRNA^GLY^ gene [64]. Disruption of other types of complexes that interact with chromatin, such as the Paf1 complex that associates with elongating RNA Pol II, causes an increase in both Ty1 mobility and Ty1 element insertion upstream of *SUF16* [64–66]. Paf1 stimulates the monoubiquitylation of histone H2B (H2B K123Ub) by the Bre1-Rad6 ubiquitin ligase complex [67]. Interestingly, genome-wide Ty1 mapping in a *rad6Δ* mutant demonstrated that Ty1 elements insert more frequently into open reading frames compared to a wild type strain [13]. An attractive hypothesis that emerges from these observations is that modification of nucleosomes by Paf1 associated Bre1-Rad6 restricts insertion of Ty1 elements. A screen for mutants that negatively regulate Ty1 transposition (*rtt* mutants) identified the Rtt109 histone acetyltransferase and Rtt106 histone chaperone [68]. Rtt109 catalyzes the acetylation of Histone H3 lysine 56 on newly synthesized H3-H4 dimers which interact with Rtt106 to promote replication coupled nucleosome assembly [69]. Stalling of DNA replication in the absence of either Rtt109 or Rtt106 may allow for increased Ty1 mobility. However, genome-wide mapping of Ty1 element insertion in an *rtt109Δ* mutant strain revealed a similar pattern to wild type strains suggesting that Rtt109 does not directly affect Ty1 targeting [13]. A complete understanding of how chromatin remodelling and histone modifications may impact Ty1 targeting and mobility will be aided by histone mutant libraries. For example, a comprehensive library of H2A and H2B mutants has been generated that could be used for testing Ty1 targeting [70]. A systematic screen of Ty1 targeting in mutants of all chromatin-modifying complexes could also be performed. Ultimately, structural studies of the Ty1 intasome in complex with nucleosomes is a critical step for understanding Ty1 element integration into the genome.

## 3-dimensional organization of tRNAs in the nucleus

The intranuclear positioning of tRNA genes could potentially affect the dynamics of Ty1 insertion. Multiple *S. cerevisiae* studies have assessed the localization of tRNA genes in the nucleus and different technical methods reveal different localization patterns. Fluorescence *in situ* hybridization demonstrated that yeast tRNA genes, although dispersed on linear chromosome maps, cluster in the nucleolus in a condensin-dependant manner [57, 71]. Chromosome conformation capture studies identified a cluster of tRNA genes that co-localized with the nucleolar ribosomal DNA (rDNA) repeats and another cluster that co-localized with centromeres [72–75]. Live-cell imaging of fluorescently labelled tRNA genes in *S. cerevisiae* demonstrated that tRNA genes can reside at the nucleolus, the nuclear periphery and in the nucleoplasm [76, 77]. In the live-cell imaging studies, the frequency of tRNA association with the nuclear periphery or nucleolus depends on how far the tRNA gene is from a tethering element such as the centromere, telomere, or rDNA. For example, *SNR6* is located close to the rDNA and exclusively localizes to the nucleolus whereas *SUP53*, which is located 23 kb from CENIII, is excluded from the nucleolus [77]. A tRNA gene with no constraints may localize to the nucleolus, nucleolar periphery or nuclear periphery [77]. Fluorescence microscopy and chromatin immunoprecipitation (ChIP) studies demonstrated that tRNA genes are recruited to the nuclear pore complex (NPC) during G2/M phase which also happens to be the peak of tRNA gene expression [78]. These studies highlight the dynamic 3-dimensional positioning of tRNA genes in the nucleus during the yeast cell cycle. Furthermore, evidence is gathering that tRNA genes have broad global effects on genome structure and organization by providing tethers to cellular structures such as the nucleolus, nuclear periphery and mitotic spindle [77–79]. Our group has recently discovered that nuclear basket proteins, which are located on the nuclear side of the NPC, are required for targeting Ty1 elements upstream of tRNA genes [80]. In the absence of the nuclear basket proteins, Ty1 elements are targeted to subtelomeric regions, similar to the Rpc40sp mutant strain described above [80]. HIV-1 viral cDNA is preferentially inserted into transcriptionally active genes that are localized near the nuclear envelope [81]. The HIV-1 intasome also localizes near the nuclear periphery and the chromatin environment on the nuclear basket side of the NPC is favourable for HIV-1 insertion [82, 83]. Chromatin that resides near the nuclear pore may therefore serve as a convenient site for intasomes to insert their cDNA immediately after passage through the NPC.

## Comparison of Ty1 and Ty3 targeting

The *S. cerevisiae* Ty3-*gypsy* retrotransposon also selectively targets genes transcribed by RNA Pol III, however, unlike Ty1, it has a precise integration site that maps to within 1–4 nucleotides of the Pol III TSS [84–86]. There are two full length Ty3 elements in the S288C *S. cerevisiae* genome and only one is active [9]. Similar to Ty1, a functional Pol III promoter is required for Ty3 transposition as mutation of the box A or box B promoter sequences prevents insertion of the Ty3 element [85, 87]. However, a tRNA gene with reduced transcriptional activity due to mutations in the transcription initiation region is still an active Ty3 target [85]. The ability of TFIIIC and TFIIIB to load onto the tRNA promoter is essential for Ty3 targeting but a wild type level of tRNA gene transcription is not. In vitro reconstitution with recombinant TFIIIB proteins demonstrated that Ty3-IN, TBP(Spt15) and Brf1 are required for Ty3 insertion while addition of the third component of TFIIIB, Bdp1, enhances integration efficiency [88, 89]. The conserved domain of TBP inserted between the N and C-terminal segments of Brf1, which can function to initiate Pol III transcription, can also mediate Ty3 insertion in vitro [90, 91]. Extra TFIIIC sites in the yeast genome that bind TFIIIC but not TFIIIB or Pol III, are not targeted by Ty3, further strengthening the argument that TFIIIB is the key Ty3 targeting factor [92, 93].

Although TFIIIB is the host factor for Ty3-IN, TFIIIC also influences the Ty3 insertion pattern. The C-terminus of Tfc1 physically interacts with Ty3-IN and enables Ty3 insertion in both orientations [88, 94]. By comparison, no physical interaction was detected between Ty1-IN and Tfc1, Tfc3 or Tfc7 in co-purification experiments from yeast lysates [30]. Another interesting difference between Ty1 and Ty3 targeting is that RNA Pol III, which is required for Ty1 element insertion, is inhibitory to Ty3 insertion in vitro [87, 95]. Genome-wide Ty1 and Ty3 insertion site mapping studies have also discovered interesting targeting differences between the two retrotransposons. For example, Ty3, unlike Ty1, does not target to nucleosomes [13, 14, 93]. Ty3 is capable of inserting at the TSS of the tRNA relic gene *ZOD1* which is bound by the Pol III machinery whereas Ty1 is not [13, 14, 93]. The lack of Ty1 targeting to *ZOD1* may be due to low *ZOD1* transcription levels [13, 14]. Interestingly, the *ZOD1* locus is activated upon nucleosome depletion which may also prevent Ty1 targeting [96]. Finally, Ty3 elements only integrate at Pol III-transcribed genes whereas Ty1 elements are capable of integrating at other genomic loci such as within silent mating cassettes, within or near Pol II-transcribed genes and at sub-telomeric regions [29, 97–102]. Ty1-IN may interact with alternative host factors to achieve insertion into such a variety of genomic regions. Although Ty1 and Ty3 are both targeted upstream of Pol III-transcribed genes, they have devised different targeting mechanisms for insertion into the genome.

## tRNA targeting TEs in other yeast species

The *Saccharomyces* sensu stricto genus includes seven natural species: *S. arboricolus,* *S. cerevisiae*, *S. eubayanus, S. kudriavzevii*, *S. mikatae,* *S. paradoxus*, *S. uvarum*, and two hybrid species: *S. pastorianus* and *S. bayanus* [103–105]. There is variation in the presence or absence of Ty elements in these species and the abundance of a particular element can vary widely between strains [106–108]. For example, Ty3 and Ty5 elements do not occur in *S. uvarum* [109]. A novel Ty3-like element, called Ty3p, was discovered in *S. paradoxus* that shares 82% nucleotide identity with an *S. cerevisiae* Ty3 element (YGRWTy3–1) and is inserted ~ 6 bp upstream of a tRNA TSS (Table 1) [110]. Degenerate solo LTRs of Ty3p are also present in the *S. cerevisiae* genome [111]. The targeting of Ty1, Ty2, Ty3, and Ty4 elements upstream of tRNA genes is conserved in the *Saccharomyces* sensu stricto genus.

**Table 1:** Retrotransposons that integrate adjacent to tRNA genes

Mobile element	Clade	Host^a^	Preferred integration site	Host factors mediating this insertion preference
LTR Retrotransposons				
Ty1	Ty1-*copia*	*Saccharomyces cerevisiae*	~ 1 kb window upstream of RNA Pol III-transcribed genes, including tRNA and 5S rRNA genes [13, 14, 21]	Ty1-IN interaction with Rpc40 [29] and Ty1-IN interaction with Rpc53, Rpc34, Rpc31 [30]
Ty2	Ty1-*copia*	*Saccharomyces cerevisiae*	~ 1 kb window upstream of RNA Pol III-transcribed genes [21]	
Ty3	Ty3-*gypsy*	*Saccharomyces cerevisiae*	1–4 bp upstream of tRNA TSS^b^ [93]	Ty3-IN interaction with TFIIIB [88, 89]
Ty3p	Ty3-*gypsy*	*Saccharomyces paradoxus*	~ 6 bp upstream of tRNA TSS [110]	
Ty4	Ty1-*copia*	*Saccharomyces cerevisiae*	~ 1 kb window upstream of RNA Pol III-transcribed genes [21]	
Tj1	Ty3-*gypsy*	*Schizosaccharomyces japonicus*	1–10 bp upstream of tRNA TSS [121]	
Beta/Tca8	Ty3-*gypsy*	*Candida albicans*	6-30 bp upstream of tRNA MCS^c^ [119]	
Tyl3	Ty3-*gypsy*	*Yarrowia lipolytica*	~ 5 bp upstream of tRNA TSS [116]	
Ylt1	Ty3-*gypsy*	*Yarrowia lipolytica*	~ 5 bp upstream of tRNA TSS [117]	
Tyl6	Ty3-*gypsy*	*Yarrowia lipolytica*	~ 5 bp upstream of tRNA TSS [115]	
DGLT-A	Ty3-*gypsy*	*Dictyostelium discoideum*	13–33 bp upstream of tRNA MCS [125]	
Skipper-2	Ty3-*gypsy*	*Dictyostelium discoideum*	8–23 bp downstream of tRNA gene [124, 172]	
Non-LTR Retrotransposons				
TRE5	L1	*Dictyostelium discoideum*	40–54 bp upstream of tRNA MCS; 37–41 bp upstream of extrachromosomal 5S rRNA genes [125, 134, 135, 174]	TRE5 ORF1 interaction with TFIIIB [132]
TRE3	L1	*Dictyostelium discoideum*	40–150 bp downstream tRNA genes [137, 175]	
NLTR-A	L1	*Dictyostelium purpureum*	2–6 bp upstream of tRNA MCS [124]	
NLTR-B	L1	*Polysphondylium pallidum*	39–64 bp upstream of tRNA MCS [124]	

^a^Host that retrotransposon was first identified in

^b^TSS refers to the tRNA transcription start site which is ~ 10 bp upstream of the mature tRNA

^c^MCS refers to the tRNA mature coding sequence

The rapid pace of whole genome sequencing in a variety of fungal species has revealed the diversity of retrotransposons [112–114]. Interestingly, a subset of these newly discovered TEs in the fungal Ascomycota phylum are distributed in the genome nearby tRNA genes (Table 1). The genome of the oleaginous yeast, *Yarrowia lipolytica* contains three Ty3-*gypsy*-like elements (Tyl3, Ylt1, Tl6) located upstream of Pol-III transcribed genes (Table 1) [115–117]. *Candida albicans* *(C. albicans)* is an opportunistic human fungal pathogen that contains 34 LTR-retrotransposon families (alpha, beta, gamma, etc.) in its genome that belong to the Ty1-*copia* and Ty3-*gypsy* families [118]. The beta LTR of the Tca8 element, which has partial elements remaining in the genome, is localized within 30 bp upstream of the mature coding sequence (MCS) of tRNA genes (Table 1) [119]. An investigation of the Pol III targets in *C. albicans* using Rpc82 ChIP-chip revealed that Rpc82 bound tRNA genes at high occupancy and retrotransposon elements at low occupancy [120]. The low occupancy binding of Rpc82 to elements such as Tca8 is likely due to amplification of Rpc82 binding to tRNA genes located adjacent to retrotransposon elements in the *C. albicans* genome [120].

Whole genome sequencing and comparison of fission yeast genomes revealed that the *Schizosaccharomyces japonicus* (*S. japonicus*) genome contains 10 families (Tj1 to Tj10) of Ty3-*gypsy* related retrotransposons clustered at the centromeres and telomeres [121, 122]. Notably, retrotransposons were dramatically reduced or lost in the other fission yeast genomes likely due to evolutionary change in control of centromere function [122]. Since tRNA genes are clustered at the centromere, the Levin lab hypothesized that the *S. japonicus* retrotransposons may be specifically targeted to tRNA genes. They tested this hypothesis by cloning the *S. japonicus* Tj1 retrotransposon and analyzing its integration behaviour in the related fission yeast *S. pombe* [121]. As predicted, the Tj1 transposon inserted 1–10 bp upstream of the TSS of tRNA genes and also at the Pol III-transcribed 5S rRNA gene (Fig. 1, Table 1) [121]. Therefore, *S. japonicus* Tj1 targets Pol III-transcribed genes and has similar insertion behaviour to Ty3 retrotransposons.

The diversity of retrotransposons in fungal species now includes Ty1-c*opia*, Ty3-g*ypsy* and LINE elements [112–114, 123]. The target specificity of each of these retrotransposons has not been fully elucidated but it is likely that Pol III-targeting will feature prominently [123].

## TEs target RNA pol III transcribed genes in *Dictyostelium*

**Fig. 3:**
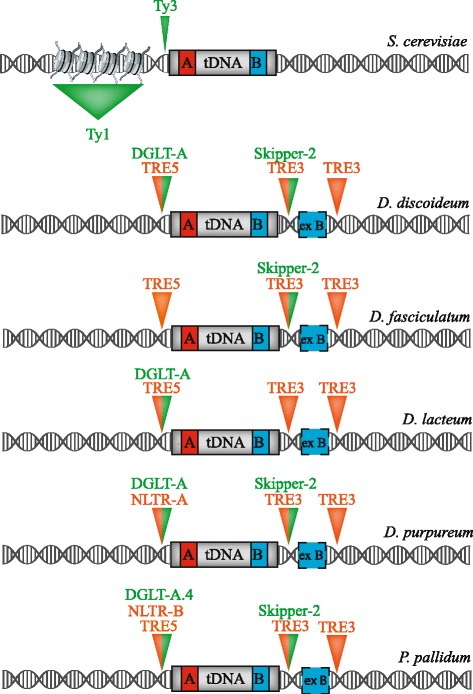
tRNA targeted retrotransposon insertion site profiles. The insertion site preference for *S. cerevisiae*, *Dictyostelium* and *P. pallidum* are shown here upstream and downstream of a tRNA gene. The tRNA gene (gray) contains box A (red) and box B (blue) internal promoters and the external box B (ex B, blue) for social amoeba. LTR-retrotransposons are in green and non-LTR retrotransposons are in orange. Inverted orange or green triangles denote retrotransposon insertion windows ranging from 2 to ~ 1000 bp upstream or 7 to ~ 450 bp downstream of the tRNA gene (not drawn to scale). For the social amoeba*,* split orange and green inverted triangles denote overlapping insertion footprints for LTR (DGLT-A, Skipper-2) and non-LTR (NLTR-A, NLTR-B, TRE5, TRE3) retrotransposons. For *P. pallidum*, a specific DLGT-A (DGLT-A.4) is indicated because DGLT-A.1–3 do not target to tRNA genes in this organism [124]. The green triangle with a broader base represents the larger insertion window for *S. cerevisiae* Ty1 which can insert up to ~ 1 kb upstream of a Pol III-transcribed gene. Nucleosomes are depicted upstream of the *S. cerevisiae* tRNA gene as Ty1 inserts into nucleosomes

Mobile elements in other organisms with compact genomes have also found a safe haven by inserting adjacent to tRNA genes; the social amoeba model organism *Dictyostelium discoideum* (*D. discoideum*) is one such organism. *D. discoideum* has tolerated an expansion of tRNA targeting retrotransposons to 3.8% of its genome whereas 0.9% or less of the genomes of other social amoeba contain tRNA associated retrotransposons [124]. It is not known what selection pressure may have allowed retroelement expansion in *D. discoideum* [124]. The *Dictyostelium gypsy*-like transposon (DGLT-A) belongs to the Ty3-*gypsy* clade of retrotransposons and preferentially inserts 13 to 33 bp upstream of the tRNA MCS in either orientation (Fig. 1, Table 1) [125]. The lack of full length DGLT-A elements in the *D. discoideum* genome suggests that they are no longer active [124]. Skipper-1 is another LTR retrotransposon in the *D. discoideum* genome that is related to DGLT-A and the Ty3-*gypsy* clade. Skipper elements, which accumulate at the centromere, contain a characteristic chromo domain (CHD) in the C-terminus of the Skipper IN protein [126]. The CHD may be important for targeting Skipper-1 into heterochromatin at the centromere [127]. Skipper-2 (previously named DGLT-P) has a diverged CHD and instead of targeting to centromeres is targeted ~ 8-23 bp downstream of tRNA genes (Fig. 3) [124]. Notably, Skipper-2 has also been identified in other amoeba species, including *Dictyostelium purpureum (D. purpureum), Dictyostelium fasciculatum (D. fasciculatum)*, and *Polysphondylium pallidum* (*P. pallidum*), where it is located ~ 140 bp downstream of tRNA genes (Table 1) [124]. Skipper-2 is the first LTR retrotransposon that preferentially integrates downstream of a tRNA gene [124]. It will be interesting to determine if the diverged CHD is responsible for targeting Skipper-2 downstream of tRNA genes.

The *D. discoideum* genome also contains non-LTR retrotransposons called TREs for tRNA gene-targeted retroelements. TRE5 elements preferentially integrate upstream (5′) of tRNA genes, whereas TRE3 elements are targeted downstream (3′) of tRNA genes; the element names are a convenient reminder of their integration preference (Figs. 1,3) [128–130]. There are three TRE5 elements (TRE5-A,B,C) and four TRE3 elements (TRE3-A,B,C,D) in the *D. discoideum* genome with TRE5-A and TRE3-A in the highest abundance [128]. TRE5 elements insert ~ 44-54 bp upstream of the tRNA MCS in the opposite transcriptional orientation (Table 1, Fig. 3) [130]. The TRE5-A retrotransposon has two ORFs - ORF1 encodes a 51kD protein of unknown function and ORF2 encodes a protein with an apurinic/apyrimidinic endonuclease (APE) domain, an RT domain, and a zinc-finger (ZF) domain (Fig. 1) [129, 131]. Interestingly, protein-protein interactions have been detected between the TRE5-A ORF1 protein and the three *D. discoideum* TFIIIB proteins TBP, Brf1 and Bdp1 [132]. Despite the similarity to Ty3, which also interacts with TFIIIB, the molecular basis of TRE5-A targeting may differ from Ty3 because of the mechanism by which TRE5-A elements integrate into the genome. Non-LTR retrotransposons such as TRE5-A elements replicate by target-primed reverse transcription whereby the APE domain nicks the target DNA which allows for reverse transcription followed by integration of the element [6]. However, similar to Ty3 elements, mutations of the box B promoter that interfere with binding of TFIIIC abolish the targeting of TRE5-A to the tRNA target gene [133]. TRE5-A insertion profiling demonstrated that TRE5-A can also integrate at the Pol III-transcribed ribosomal 5S gene which is located on a multi-copy extrachromosomal DNA element harboring the rRNA genes [134, 135]. Unlike TRE5, TRE3 has a broader range of insertion that is 40–150 bp downstream of tRNA genes in the same transcription orientation (Fig. 3) [130]. The broader insertion window is because TRE3 can target downstream of either the tRNA internal box B or an external box B (ex B) that is positioned ~ 100 bp downstream of the internal box B and present at ~ 80% of *D. discoideum* tRNA genes (Fig. 3) [136, 137]. New non-LTR retrotransposons (NLTR) were recently identified in the genomes of *D. purpureum* (NTLR-A) and *P. pallidum* (NLTR-B) that are distantly related to TRE elements [124]. *P. pallidum* NLTR-B inserts upstream of tRNA genes in a similar manner to TRE5 elements however *D. purpureum* NLTR-A has a unique insertion specificity 2-6 bp upstream of the tRNA MCS (Fig. 3) [124].

## Evolutionary selection of pol III transcribed genes as a genomic target for insertion

Survival of mobile elements in the compact *Saccharomyces* and *Dictyostelium* genomes necessitated insertion of the element in a locus that minimized host genome damage [138]. During evolution, retrotransposons have independently developed targeting to tRNA genes at least six times in dictyostelids and at least four times (Ty1–4) in *S. cerevisiae* [124]. Insertion upstream of Pol III-transcribed genes has the advantage that most Pol III-transcribed genes exist in multiple copies, therefore they are an abundant target and insertion into one locus is not likely to be lethal. Furthermore, the promoter elements of tRNA genes are embedded within the coding region and inserting upstream of tRNA genes will not damage promoter activity. The *S. cerevisiae* genome has 275 copies of tRNA genes for decoding the 20 standard amino acids, and the 5S rRNA exists in a tandem array consisting of 100–200 copies [8]. Therefore, there are plenty of target sites available for Ty1–4 retrotransposon integration. *D. discoideum* and *D. purpureum* have an expansion in the number of their tRNA genes (418 and 353, respectively) compared to other dictyostelids [124]. The large number of tRNA genes has allowed amplification of the DGTL-A retrotransposon in *D. discoideum* but not the other dictyostelids, including *D. purpureum* [124]. Therefore, an increase in the target site, in this case a tRNA gene, does not always give a retrotransposon freedom to increase in abundance [124]. Insertion of retrotransposons downstream of tRNA genes is only found in dictyostelid genomes (TRE3 and Skipper-2) but not in the *S. cerevisiae* genome [124]. Integration of retrotransposons downstream of *S. cerevisiae* tRNA genes may negatively impact tRNA or adjacent gene transcription and overall cell fitness. The insertion of Ty1 or Ty3 elements upstream of tRNA genes does not appear to negatively affect tRNA gene transcription in *S. cerevisiae*. On the contrary, evidence shows that these elements have a neutral or moderately stimulatory effect on tRNA gene transcription [139, 140]. It has not yet been investigated if tRNA gene expression is affected in *D. discoideum* when retrotransposons insert nearby [131]. The retrotransposon may benefit however from its targeting preference because the promoter activity of the A module in TRE5-A is enhanced if a tRNA gene is present upstream [141].

Whether or not Ty1 insertion events are advantageous or deleterious to the cell has no simple answer. Single novel Ty1 insertions upstream of Pol III-transcribed genes have no growth advantage or disadvantage compared to a parental strain lacking Ty insertions [142]. This data is consistent with the theory that the insertion site of Ty1 elements has evolved to minimize deleterious effects on the host genome [142]. Ty1 elements also have an internal mechanism of copy number control which likely evolved to prevent retrotransposon bursts that decrease host cell fitness due to genome instability. Expression from an internal promoter of a protein derived from the C-terminal half of Gag inhibits retrotransposition in a dose-dependent manner [143, 144]. Ty1 transposition must be artificially induced to assess the effect of increased Ty1 copy number. As the copy number of novel Ty1 elements doubles, yeast strains develop a wide range of growth phenotypes including insertions that do not affect strain growth, those that confer a negative fitness effect and those that confer a growth advantage [145, 146]. Remarkably, Ty1 copy number can be increased as much as 10-fold and still only modest growth phenotypes are detected [147]. However, with a 10-fold increase in Ty1 elements, the strains become highly sensitive to DNA damaging agents due to increased ectopic recombination [147].

## Mechanisms of Ty1-mediated genome evolution

Ty elements can cause genome evolution by a variety of mechanisms [148]. If transcription of the Ty1 element is induced, for example in response to environmental stress (UV light, ionizing radiation) then Ty1-IN mediated insertion events may be a mechanism of genome evolution [149–151]. DNA replication stress, DNA damage and genome damage due to telomere erosion can also activate Ty1 mobility [152–154]. Increased Ty1 mobility is also responsible for chromosome rearrangements in aging yeast populations [155]. Induction of Ty1 transcription and transposition under stress is thought to be a strategy to increase cell survival by inducing adaptive mutations. Ty1 predominantly inserts upstream of Pol III-transcribed genes but can also insert into Pol II-transcribed genes or in subtelomeric regions [13, 14, 29, 80]. Insertion of Ty1 into the *URA3* gene can be detected when cells are grown on 5-Fluoroorotic acid which is toxic to cells unless the *URA3* locus is mutated and cells are supplemented with uracil [98]. Another classic example of Ty1 insertion into a Pol II-transcribed gene is mutation of the *CAN1* locus which results in resistance to the arginine analogue canavanine [102].

Repetitive elements such as Ty retrotransposons and tRNA genes are fragile genomic sites because they are prone to genome rearrangement. Experimental evolution of *S. cerevisiae* in a glucose-limited environment caused chromosomal rearrangements due to ectopic recombination between tRNA genes, entire Ty elements or solo LTRs on different chromosomes [156]. Double-strand breaks (DSBs) induced by ionizing radiation or perturbations of essential DNA replication proteins cause chromosome breakage at repetitive Ty elements and chromosome translocations due to ectopic recombination with Ty elements on other chromosomes [157–161]. DSBs can also be repaired by ectopic recombination using Ty elements that are located up to ~ 50 kb away from the break site [162]. Interestingly, DSB repair has also been shown to occur at NPCs, where active transcription tRNA genes occurs [163, 164]. Pol III-transcribed genes are also prone to RNA:DNA hybrid formation (R-loops) that are susceptible to DNA damage due to exposure of single stranded DNA [165, 166]. In the absence of RNAse H, which removes RNA:DNA hybrids, Ty1 cDNA also forms R-loops likely during reverse transcription, and is elevated ~ 3-fold resulting in increased Ty1 mobility [166]. Taken together, tRNA and Ty repetitive elements are dynamic regions of genetic movement contributing to the evolutionary flux of the eukaryotic genome.

## Conclusions

Retrotransposons and retroviruses have successfully utilized the Pol III transcription machinery and Pol III-transcribed genes to replicate in eukaryotic cells. Both retrotransposons and retroviruses use a tRNA priming system for reverse transcription. SINE elements, which constitute ~ 11% of the human genome, evolved from tRNA priming of retroviral genomes and contain box A and box B elements in their 5′ regions [167, 168]. Both yeast and social amoeba retrotransposons with different structures and ORFs have found a safe haven near tRNA genes (Fig. 3). The ongoing search for new TEs that are targeted adjacent to Pol III-transcribed genes and the host factors required for their insertion will allow better understanding of the mechanisms used by retrotransposons and retroviruses to gain access to host genomes. Future studies on how mobile elements contribute to the maintenance of the global architecture of the genome will provide novel evolutionary insights into the importance of these abundant elements.

## Abbreviations

APE: Apurinic/apyrimidinic endonuclease
*C. albicans*: *Candida albicans*
ChIP: Chromatin immunoprecipitation
*D. discoideum*: *Dictyostelium discoideum*
DGLT-A: *Dictyostelium gypsy-*like transposon
DSB: Double-strand break
ex B: External box B
IN: Integrase
IP: Immunoprecipitation
LINE: Long interspersed element
LTR: Long terminal repeat
MCS: Mature coding sequence
mRNA: Messenger RNA
NLTR: Non-LTR retrotransposon
NPC: Nuclear pore complex
ORF: Open reading frame
*P. pallidum*: *Polysphondylium pallidum*
PFV: Prototype foamy virus
Pol: Polymerase
PR: Protease
rDNA: Ribosomal DNA
RH: Ribonuclease H
rRNA: Ribosomal RNA
RT: Reverse transcriptase
*S.cerevisiae*: *Saccharomyces cerevisiae*
*S.japonicus*: *Schizosaccharomyces japonicus*
*S.pombe*: *Schizosaccharomyces pombe*
SINEs: Short interspersed elements
TBP: TATA binding protein
TE: Transposable elements
TOR: Target of rapamycin
TRE: tRNA gene targeted retroelement
tRNA: Transfer RNA
TSS: Transcription start site
UTR: Untranslated region
VLP: Virus-like particle
ZF: Zinc-finger
